# Supplementary material for: Extensive substrate recognition by the streptococcal antibody-degrading enzymes IdeS and EndoS
Source: Nat Commun. 2022 Dec 17;13:7801. doi: 10.1038/s41467-022-35340-z (PMC9759587; doi:10.1038/s41467-022-35340-z)
Supplement: Supplementary file 1 — Supplementary Information [file 41467_2022_35340_MOESM1_ESM.pdf]

Supplementary information for:

Extensive substrate recognition by the streptococcal antibody-degrading enzymes IdeS and EndoS

Abigail S. L. Sudol[1], John Butler[1], Dylan P. Ivory[1], Ivo Tews[1]*, Max Crispin[1]*.

[1]School of Biological Sciences, University of Southampton, Southampton, SO17 1BJ, UK

*Correspondence to Ivo Tews (Ivo.Tews@soton.ac.uk) and Max Crispin (Max.Crispin@soton.ac.uk)

Supplementary Table 1: Crystallographic data collection and refinement statistics for less-crystallisable IgG1 "Fx" E382S mutant. Values for the highest resolution shell are shown in parentheses.

**Data Collection**	
Beamline	ID30A-3 (European Synchrotron Radiation Facility)
Resolution range (Å)	47.5-3.04 (3.25-3.04)
Space group	$P3_221$
Unit cell dimensions:<br>*a, b, c* (Å)<br>α, β, γ (degrees)	 106.78, 106.78, 104.01<br>90, 90, 120
Wavelength (Å)	0.968
Unique reflections	13476 (2358)
Completeness (%)	99.7 (98.4)
R_{merge}	0.034 (1.117)
R_{meas}	0.048 (1.580)
R_{pim}	0.034 (1.117)
$I/\sigma(I)$	15.2 (0.6)
Multiplicity	1.9 (1.9)
CC half	0.999 (0.321)
Wilson *B* factor (Å^2)	113.7
**Refinement**	
Number of reflections (all/free)	13476/654
R_{work} (%)	19.7
R_{free} (%)	21.8
RMSD[1]:<br>Bonds (Å)<br>Angles (degrees)	 0.0025<br>0.797
Molecules per ASU[2]	1
Atoms per ASU[2]	3,549
Average *B* factors (Å^2)<br>(protein/ligand/water)	 (141.27/176.11/105.09)
Model quality (Ramachandran plot):<br>Most favoured region (%)<br>Allowed region (%)<br>Outliers (%)	 95.88<br>3.63<br>0.48

[1] RMSD, root-mean-squared deviation
[2] ASU, asymmetric unit

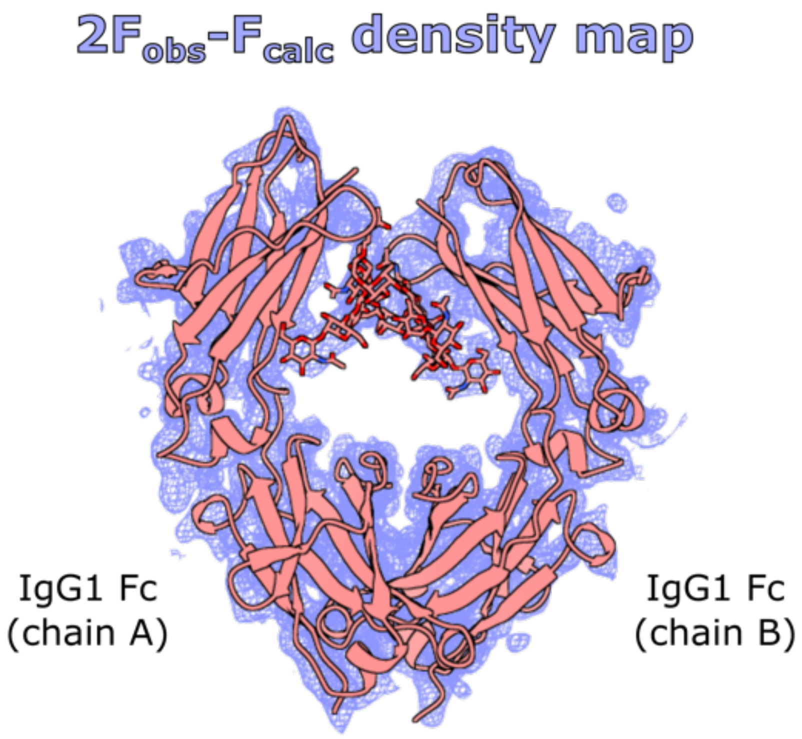

Supplementary Fig. 1: Electron density map for the refined IgG1 Fc^{E382S} model. IgG1 Fc^{E382S} is depicted in coral and as a cartoon; N-linked glycans are depicted as sticks and coloured by heteroatom (oxygen in red; nitrogen in blue). Final $2F_o$–F_{calc} map is displayed at a contour level of 1.1 σ.

Supplementary Table 2: Crystallographic data collection and refinement statistics for IdeS^{C94A}-IgG1 Fc^{E382A} complex. Values for the highest resolution shell are shown in parentheses.

**Data Collection**	
Beamline	ID30A-3 (European Synchrotron Radiation Facility)
Resolution range (Å)	48.53 – 2.34 (2.38-2.34)
Space group	*C* 1 2 1
Unit cell dimensions:<br>*a, b, c* (Å)<br>α, β, γ (degrees)	 217.56, 108.45, 63.05<br>90.00, 90.06, 90.00
Wavelength (Å)	0.9677
Unique reflections	60351 (3005)
Completeness (%)	97.7 (98.8)
R_{merge}	0.229 (0.877)
R_{meas}	0.225 (0.978)
R_{pim}	0.112 (0.426)
$I/\sigma(I)$	5.3 (1.0)
Multiplicity	4.96 (4.98)
CC half	0.954 (0.335)
Wilson *B* factor (Å^2)	34.8
**Refinement**	
Number of reflections (all/free)	60350/3049
R_{work} (%)	18.2
R_{free} (%)	20.6
Twinning fraction	0.507 for *h,k,l*<br>0.493 for *-h,-k,l*
RMSD[1]:<br>Bonds (Å)<br>Angles (degrees)	 0.0079<br>1.36
Molecules per ASU[2]	2
Atoms per ASU[2]	6,185
Average *B* factors (Å^2)<br>(protein/ligand/water)	 (38.48/45.71/38.81)
Model quality (Ramachandran plot):<br>Most favoured region (%)<br>Allowed region (%)<br>Outliers (%)	 96.95<br>2.77<br>0.28

[1] RMSD, root-mean-squared deviation
[2] ASU, asymmetric unit

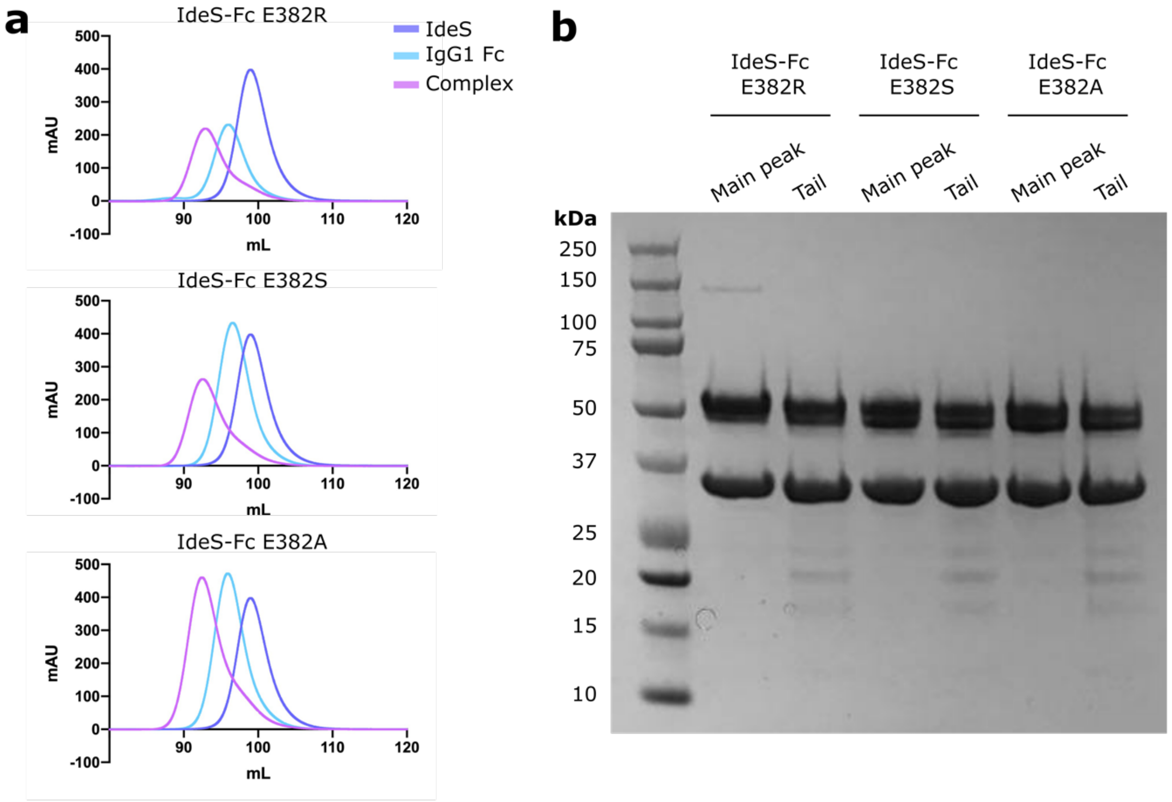

Supplementary Fig. 2: Purification of IgG1 Fc-IdeS^{C94A} complexes. a Analytical size exclusion chromatography (SEC) of IdeS^{C94A}-IgG1 Fc complexes. IdeS^{C94A} and Fcs containing E382R, E382S and E382A mutations were first applied to a Superdex 16/600 s200 column, then complexes were combined in an approximately 1:1 molar ratio and applied to the column again. **b** SDS-PAGE of the main peak and tail fractions following purification of each complex. Main peaks for each complex contain dominant bands for IgG1 Fc (~50 kDa) and IdeS^{C94A} (~34 kDa). Main peak fractions only were taken forward for crystallisation. These experiments represent the single batches of protein complexes used for the crystallisation experiments.

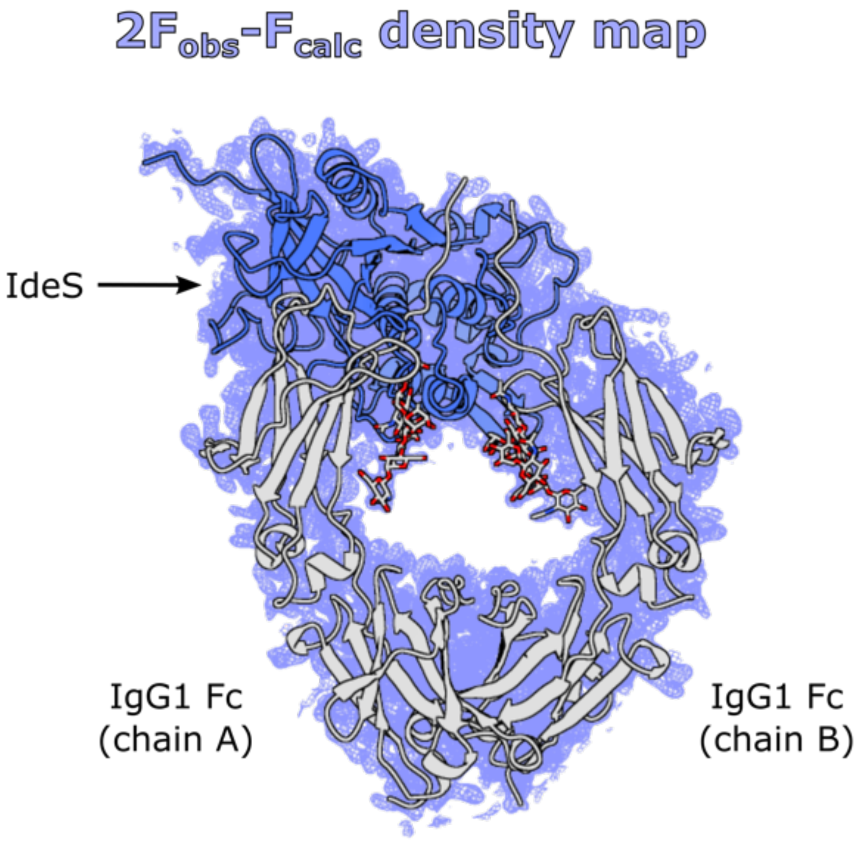

Supplementary Fig. 3: Electron density map for the refined $\text{IdeS}^{\text{C94A}}$-IgG1 Fc^{E382A} model. $\text{IdeS}^{\text{C94A}}$ is coloured blue; Fc^{E382A} is coloured silver. $\text{IdeS}^{\text{C94A}}$ and Fc^{E382A} are depicted as a cartoon; N-linked glycans on the Fc are depicted as sticks and coloured by heteroatom (oxygen in red; nitrogen in blue). Final $2F_{\text{obs}}–F_{\text{calc}}$ map is displayed at a contour level of 1.1 σ.

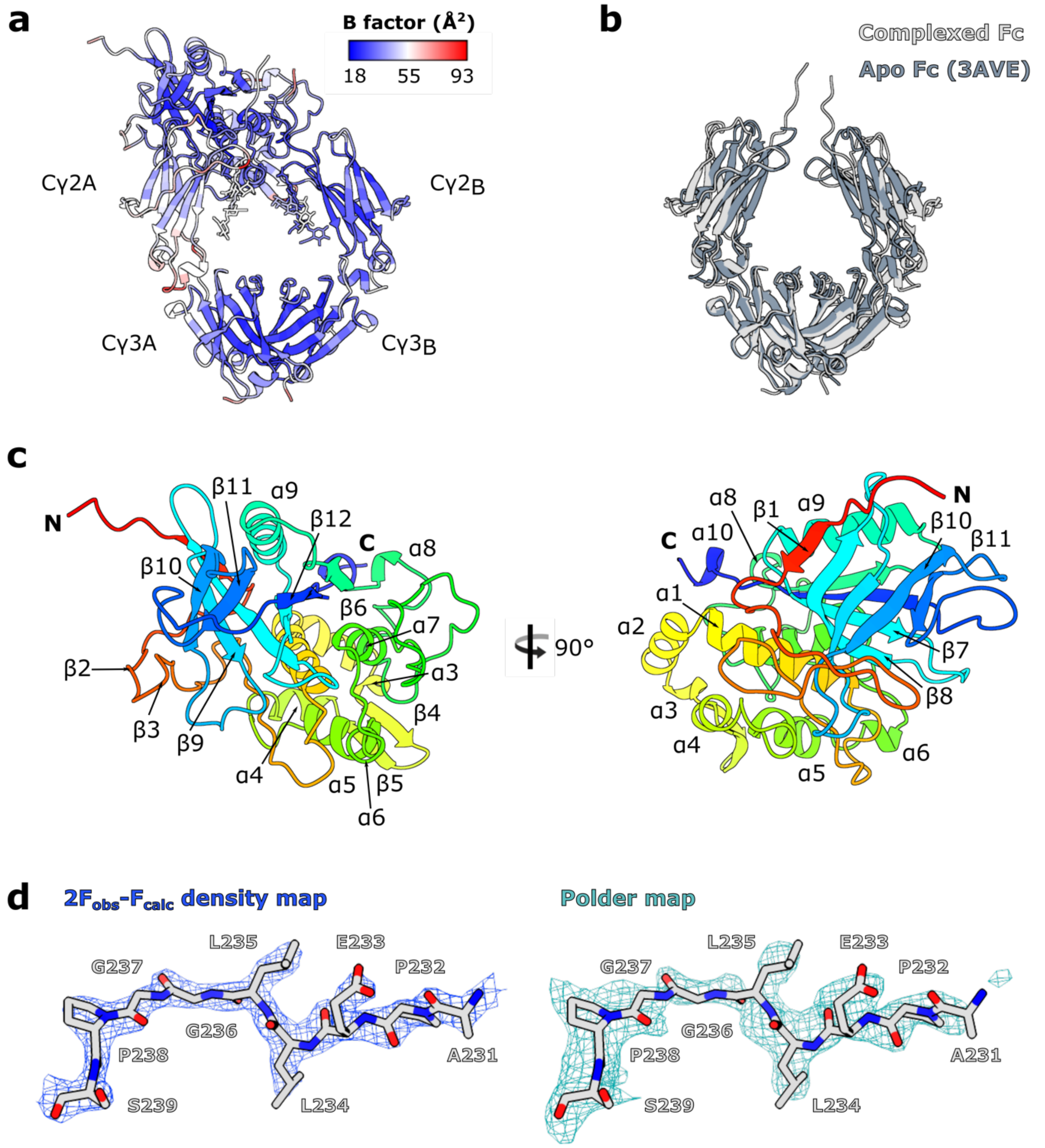

Supplementary Fig. 4: Analysis of IdeS^{C94A}-IgG1 Fc^{E382A} crystal structure. a Distribution of average *B* factors per residue for IdeS^{C94A}-IgG1 Fc^{E382A} complex. **b** Superposition of complexed IgG1 Fc^{E382A} (depicted in silver) with wild-type, apo IgG1 Fc (PDB code 3AVE; depicted in dark grey). **c** Front and side views of complexed IdeS^{C94A}, coloured as a rainbow from the N- (red) to C-terminus (blue) and labelled with secondary structure, as calculated by DSSP[1,2]. **d** Final $2F_{obs}$–F_{calc} electron density map (blue, weighted at 1.5 σ), and polder map calculated in *PHENIX*[3,4] (teal, weighted at 3 σ) for the IgG1 Fc^{E382A} hinge region (residues 231-239 in chain A) bound within the IdeS^{C94A} active site. Fc^{E382A} hinge peptide is depicted as sticks and coloured silver, with oxygen atoms coloured red and nitrogen atoms coloured blue.

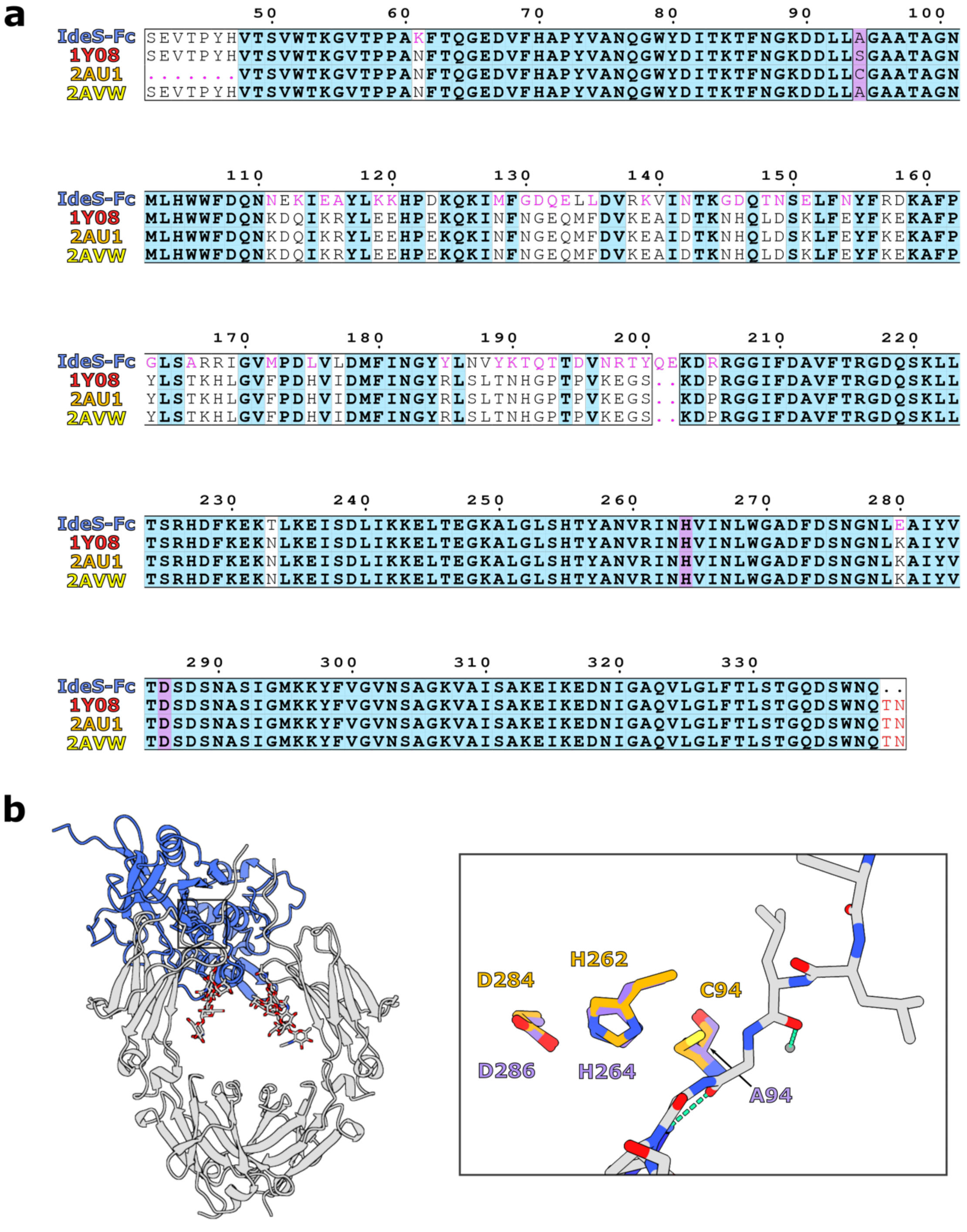

Supplementary Fig. 5: Analysis of IdeS^{C94A}-IgG1 Fc^{E382A} crystal structure (continued). a Multiple sequence alignment of complexed IdeS^{C94A} vs published apo IdeS structures (PDB codes 1Y08, 2AU1 and 2AVW; all Mac-1 variants), generated in Clustal Omega[5] and depicted using ESPript 3.0[6]. Conserved residues are coloured blue; catalytic triad residues are highlighted in purple; similar residues are shown in black font; residues with differing chemical properties are coloured in pink font. Numbering is shown for the complexed form of IdeS^{C94A} (Mac-2). **b** Superposition of catalytic residues of complexed IdeS^{C94A} vs apo wild-type IdeS (PDB code 2AU1, coloured in orange). IgG1 Fc^{E382A} is coloured silver; N-linked glycans and residues in zoom panel are coloured by heteroatom (oxygen in red; nitrogen in *blue). IdeS^{C94A} is coloured blue, catalytic residues in zoom panel are coloured purple.*

Supplementary Table 3: Crystallographic data collection and refinement statistics for EndoS$^{D233A/E235L}$-IgG1 Fc^{E382R} complex. Values for the highest resolution shell are shown in parentheses.

**Data Collection**	
Beamline	I03 (Diamond Light Source)
Resolution range (Å)	49.78-3.45 (3.51-3.45)
Space group	$P2_12_12_1$
Unit cell dimensions:<br>*a, b, c* (Å)<br>α, β, γ (degrees)	 96.529, 174.294, 193.059<br>90, 90, 90
Wavelength (Å)	0.9763
Unique reflections	43678 (2124)
Completeness (%)	100 (100)
R_{merge}	0.144 (1.951)
R_{meas}	0.150 (2.023)
R_{pim}	0.040 (0.532)
$I/\sigma(I)$	11.2 (0.6)
Multiplicity	13.7 (14.3)
CC half	1.000 (0.599)
Wilson *B* factor (Å^2)	116.4
**Refinement**	
Number of reflections (all/free)	43613/2116
R_{work} (%)	25.3
R_{free} (%)	31.1
RMSD[1]:<br>Bonds (Å)<br>Angles (degrees)	 0.0018<br>0.604
Molecules per ASU[2]	3
Atoms per ASU[2]	17087
Average *B* factors (Å^2)<br>(protein/carbohydrate/water)	157, 156, 90
Model quality (Ramachandran plot):<br>Most favoured region (%)<br>Allowed region (%)<br>Outliers (%)	 92.65<br>6.33<br>1.02

[1] RMSD, root-mean-squared deviation
[2] ASU, asymmetric unit

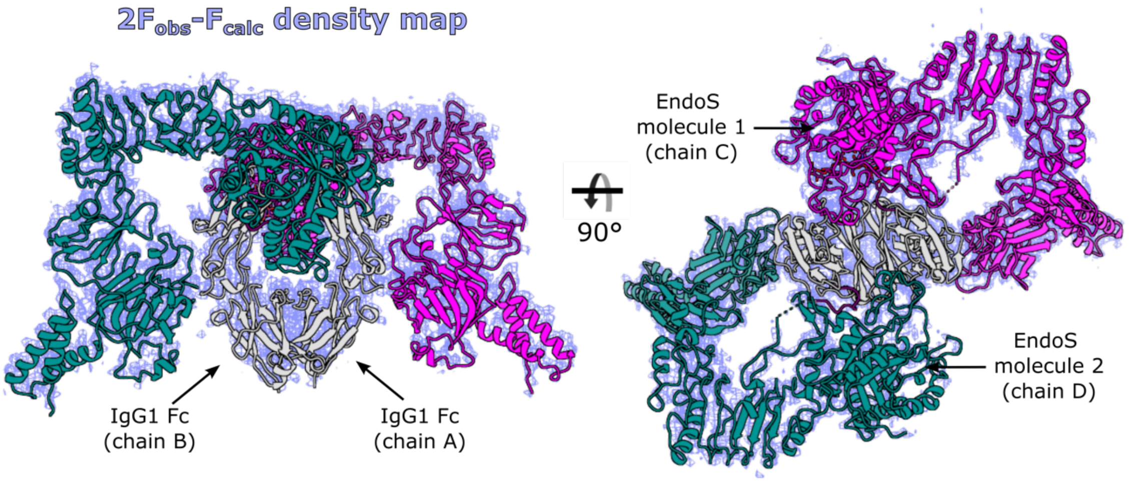

Supplementary Fig. 6: Electron density map for the refined EndoS$^{D233A/E235L}$-IgG1 Fc^{E382R} model. IgG1 Fc^{E382R} is depicted as a cartoon and coloured silver; C′E loops are coloured purple; N-linked glycans are depicted as sticks and coloured by heteroatom (oxygen in red and nitrogen in blue). EndoS molecules 1 and 2 are depicted as cartoons and coloured magenta and teal, respectively. Final $2F_o$–F_{calc} map is displayed at a contour level of 1.1 σ.

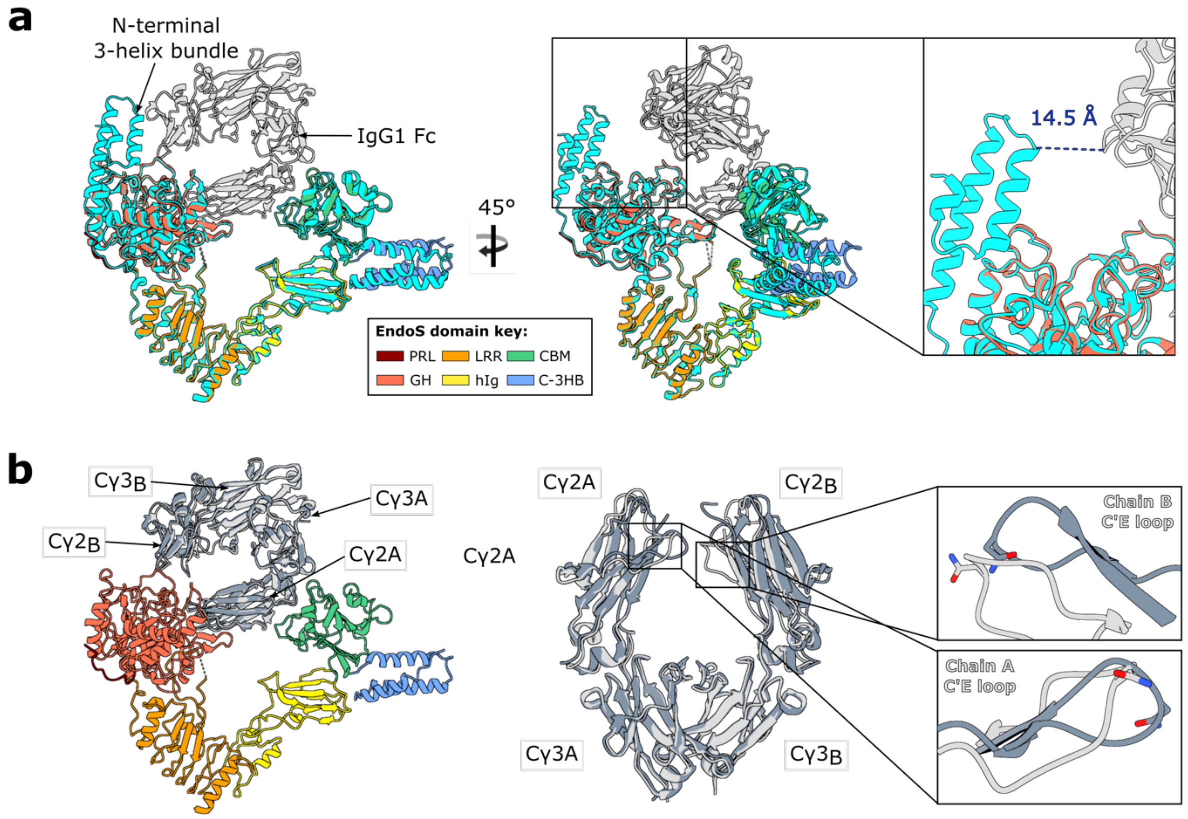

Supplementary Fig. 7: Superposition of EndoS$^{D233A/E235L}$-IgG1 Fc^{E382R} complex with respective apo structures of each component. a Superposition of full-length EndoS (PDB code 6EN3, coloured in cyan) with complexed EndoS$^{D233A/E235L}$. Focused view of N-terminal 3-helix bundle (not present in the complexed EndoS$^{D233A/E235L}$ construct) indicates that it would not bind IgG1 Fc. **b** Superposition of wild-type IgG1 Fc (PDB code 3AVE; coloured in dark grey) with complexed IgG1 Fc^{E382R}, which reveals conformational changes in the C′E loops containing the N-linked glycans at N297 (whose side chain is shown as sticks and coloured by heteroatom). **a, b** Complexed EndoS$^{D233A/E235L}$ and IgG1 Fc^{E382R} are coloured as in Fig. 4a. N-linked glycans have been omitted for clarity.

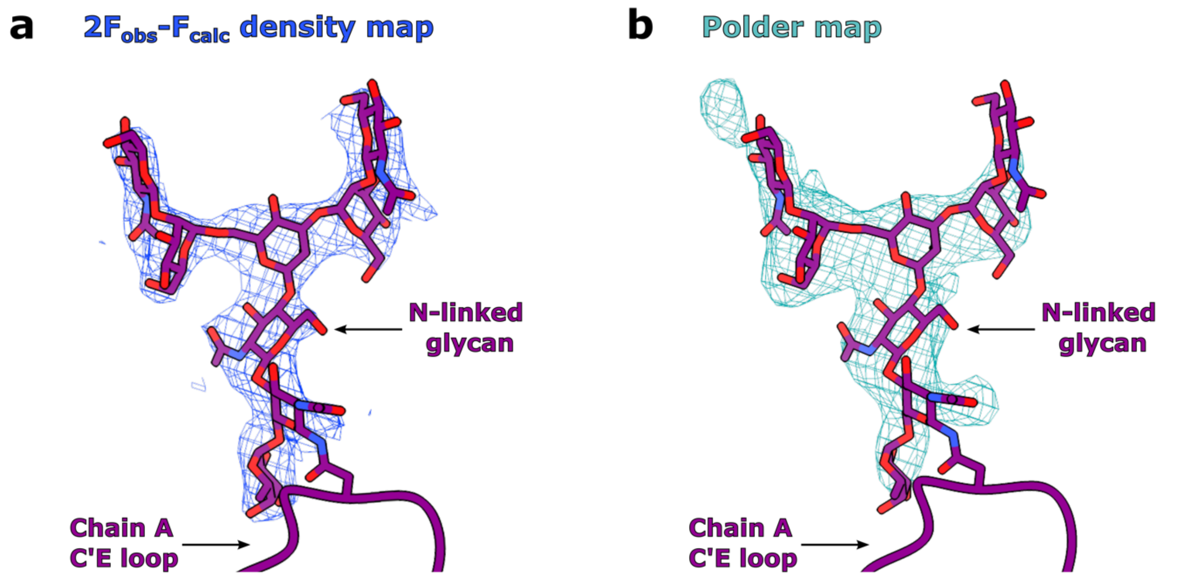

Supplementary Fig. 8: Electron density maps for the N-linked glycan on IgG1 Fc^{E382R} in complex with EndoS$^{D233A/E235L}$. a Final $2F_{obs}–F_{calc}$ electron density map (blue, weighted at 1.1 σ) for the N-linked glycan within chain A of IgG1 Fc^{E382R}. **b** Polder map for the N-linked glycan (teal, weighted at 3.5 σ), as calculated with the *phenix.polder* tool[4] within the *PHENIX* software suite[3]. A model of the EndoS$^{D233A/E235L}$-IgG1 Fc^{E382R} complex was generated by molecular replacement in Molrep[7], using models devoid of a glycan in this conformation (4NUY for EndoS; 3AVE for IgG1 Fc), and subsequently used in calculation of the polder map. **a, b** The glycan and the N297 side chain are depicted as sticks and coloured by heteroatom (oxygen in red and nitrogen in blue); the C′E loop is depicted as a cartoon.

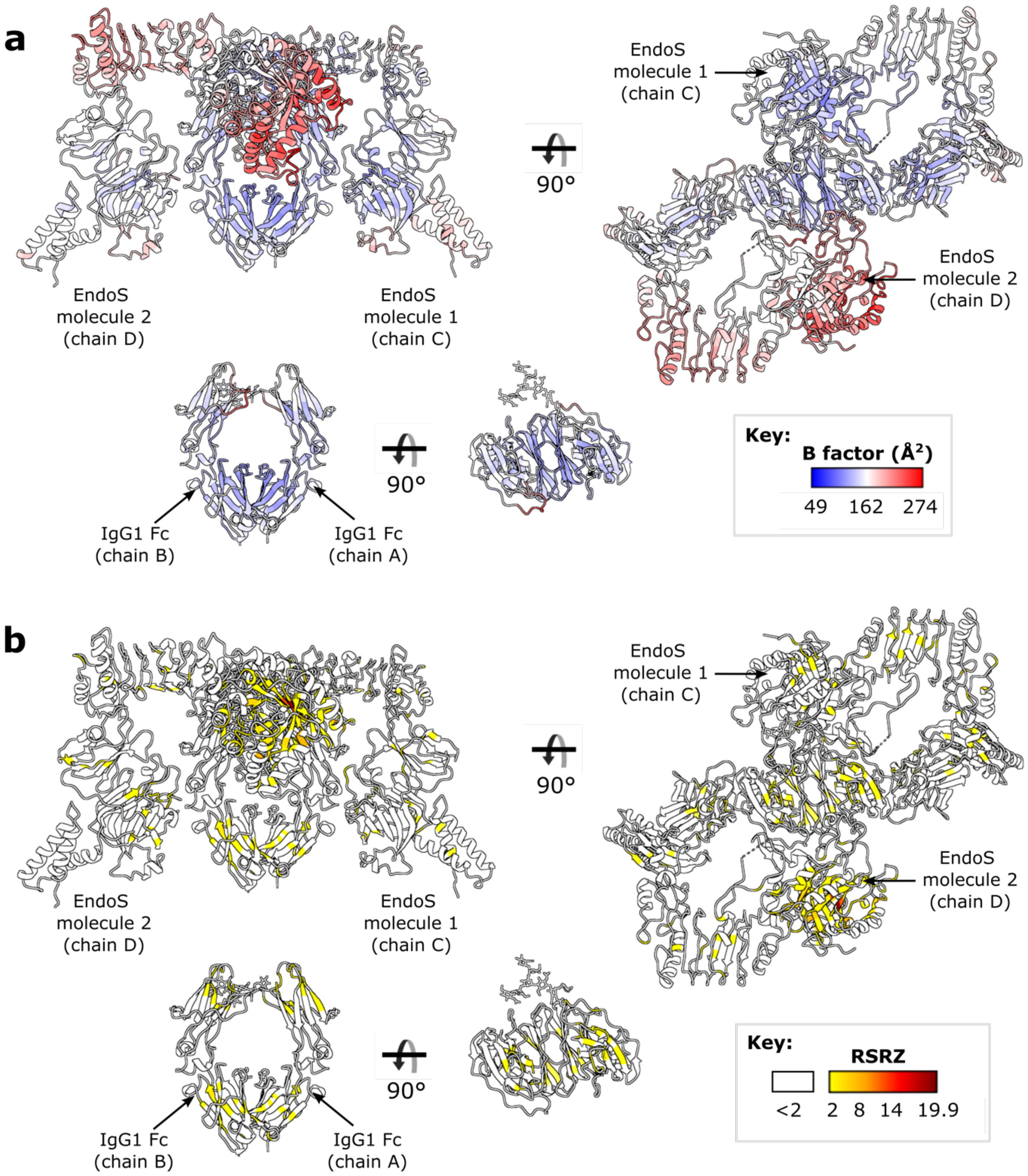

Supplementary Fig. 9: Analysis of order within the EndoS$^{D233A/E235L}$-IgG1 Fc^{E382R} crystal structure. **a** *B* factor distribution (depicted as average *B* factor per residue); **b** RSRZ distribution as reported in the validation report for this structure (PDB code 8A49). Residues identified as outliers (RSRZ of 2.0 or greater) are coloured as shown in the scale; residues not identified as outliers (RSRZ lower than 2.0) are coloured white.

Supplementary Table 4: Primers used for site-directed mutagenesis of IgG1 Fc constructs. Primers are written 5′ to 3′.

IgG1 Fc construct	Mutagenic primer sequence (5′ → 3′)	
	Forward	Reverse
E382R	GACATCGCCGTGGAGTGGAGTGGAG GAGCAATGGGCAGCCGGAGAACAAC	TTGTTCTCCGGCTGCCCATTGCTCCTCC ACTCCACGGCGATGTCG
E382S	GACATCGCCGTGGAGTGGAGTGGAG CAGCAATGGGCAGCCGGAGAACAAC	TTGTTCTCCGGCTGCCCATTGCTGCTCC ACTCCACGGCGATGTCG
E382A	GACATCGCCGTGGAGTGGAGTGGGC GAGCAATGGGCAGCCGGAGAACAAC	TTGTTCTCCGGCTGCCCATTGCTCGCC CACTCCACGGCGATGTCG

a Inactive IdeS (residues 41-339)

MSEVTPYHVTSVWTKGVTPPAKFTQGEDVFHAPYVANQG
WYDITKTFNGKDDLLAGAATAGNMLHWWFDQNNEKIEAY
LKKHPDKQKIMFGDQELLDVRKVINTKGDQTNSELFNYFR
DKAFPGLSARRIGVMPDLVLDMFINGYYLNVYKTQTTDVNR
TYQEKDRRGGIFDAVFTRGDQSKLLTSRHDFKEKTLKEISD
LIKKELTEGKALGLSHTYANVRINHVINLWGADFDSNGNLE
AIYVTDSDSNASIGMKKYFVGVNSAGKVAISAKEIKEDNIG
AQVLGLFTLSTGQDSWNQLEHHHHHH

b Inactive EndoS (residues 98-995)

MIPEKIPMKPLHGPLYGGYFRTWHDKTSDPTEKDKVNSMG
ELPKEVDLAFIFHDWTKDYSLFWKELATKHVPKLNKQGTRV
IRTIPWRFLAGGDNSGIAEDTSKYPNTPEGNKALAKAIVDE
YVYKYNLDGLDVAVLHDSIPKVDKKEDTAGVERSIQVFEEI
GKLIGPKGVDKSRLFIMDSTYMADKNPLIERGAPYINLLLVQ
VYGSQGEKGGWEPVSNRPEKTMEERWQGYSKYIRPEQYM
IGFSFYEENAQEGNLWYDINSRKDEDKANGINTDITGTRAE
RYARWQPKTGGVKGGIFSYAIDRDGVAHQPKKYAKQKEFK
DATDNIFHSDYSVSKALKTVMLKDKSYDLIDEKDFPDKALR
EAVMAQVGTRKGDLERFNGTLRLDNPAIQSLEGLNKFKKLA
QLDLIGLSRITKLDRSVLPANMKPGKDTLETVLETYKKDNK
EEPATIPPVSLKVSGLTGLKELDLSGFDRETLAGLDAATLTSL
EKVDISGNKLDLAPGTENRQIFDTMLSTISNHVGSNEQTVK
FDKQKPTGHYPDTYGKTSLRLPVANEKVDLQSQLLFGTVTN
QGTLINSEADYKAYQNHKIAGRSFVDSNYHYNNFKVSYEN
YTVKVTDSTLGTTTDKTLATDKEETYKVDFFSPADKTKAVH
TAKVIVGDEKTMMVNLAEGATVIGGSADPVNARKVFDGQL
GSETDNISLGWDSKQSIIFKLKEDGLIKHWRFFNDSARNP
ETTNKPIQEASLQIFNIKDYNLDNLLENPNKFDDEKYWITVD
TYSAQGERATAFSNTLNNITSKYWRVVFDTKGDRYSSPVVP
ELQILGYPLPNADTIMKTVTTAKELSQQKDKFSQKMLDELKI
KEMALETSLNSKIFDVTAINANAGVLKDCIEKRQLLKKLLEH
HHHHH

Supplementary Fig. 10: Constructs for inactive bacterial enzymes. a Truncated IdeS (amino acids 41-339) with a C94A mutation to abolish catalytic activity (residue A94 is highlighted in green). **b** Truncated EndoS (amino acids 98-995), with D233A/E235L mutations to abolish catalytic activity (residues A233-L235 are highlighted in green). **a, b** Initiating methionines are highlighted in yellow; C-terminal linker and his-tag are highlighted in blue.

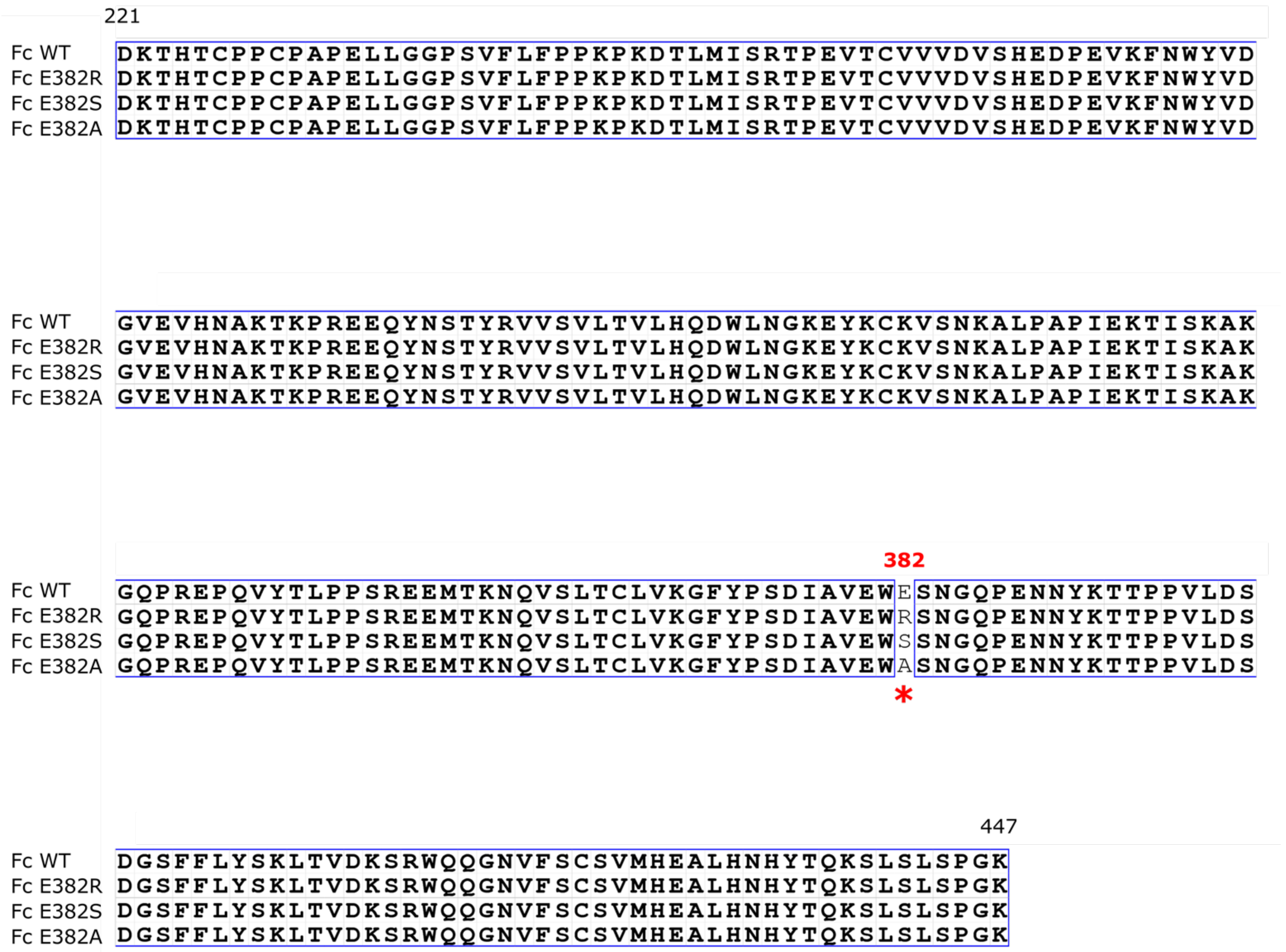

Supplementary Fig. 11: IgG1 Fc constructs expressed from pFUSE-hIgG1-Fc vectors. pFUSE-hIgG1-Fc vector expresses IgG1 Fc residues 221-447. E382R/S/A mutations introduced with site-directed mutagenesis are indicated with a red asterisk. Sequences were aligned with Clustal Omega[5] and the resulting alignment depicted using ESPript 3.0[6].

Supplementary references

1 Kabsch, W. & Sander, C. Dictionary of protein secondary structure: pattern recognition of hydrogen-bonded and geometrical features. *Biopolymers* **22**, 2577-2637, doi:10.1002/bip.360221211 (1983).

2 Touw, W. G. *et al.* A series of PDB-related databanks for everyday needs. *Nucleic Acids Res* **43**, D364-368, doi:10.1093/nar/gku1028 (2015).

3 Liebschner, D. *et al.* Macromolecular structure determination using X-rays, neutrons and electrons: recent developments in Phenix. *Acta Crystallogr D Struct Biol* **75**, 861-877, doi:10.1107/S2059798319011471 (2019).

4 Liebschner, D. *et al.* Polder maps: improving OMIT maps by excluding bulk solvent. *Acta Crystallogr D Struct Biol* **73**, 148-157, doi:10.1107/S2059798316018210 (2017).

5 Sievers, F. *et al.* Fast, scalable generation of high-quality protein multiple sequence alignments using Clustal Omega. *Mol Syst Biol* **7**, 539, doi:10.1038/msb.2011.75 (2011).

6 Robert, X. & Gouet, P. Deciphering key features in protein structures with the new ENDscript server. *Nucleic Acids Res* **42**, W320-324, doi:10.1093/nar/gku316 (2014).

7 Vagin, A. & Teplyakov, A. Molecular replacement with MOLREP. *Acta Crystallogr D Biol Crystallogr* **66**, 22-25, doi:10.1107/S0907444909042589 (2010).
