# Supplementary material for: Extensive substrate recognition by the streptococcal antibody-degrading enzymes IdeS and EndoS
Source: Nat Commun. 2022 Dec 17;13:7801. doi: 10.1038/s41467-022-35340-z (PMC9759587; doi:10.1038/s41467-022-35340-z)
Supplement: Supplementary file 3 — Reporting Summary [file 41467_2022_35340_MOESM3_ESM.pdf]

nature portfolio

Corresponding author(s): Max Crispin, Ivo Tews

Last updated by author(s): 22-11-2022

Reporting Summary

Nature Portfolio wishes to improve the reproducibility of the work that we publish. This form provides structure for consistency and transparency in reporting. For further information on Nature Portfolio policies, see our Editorial Policies and the Editorial Policy Checklist.

Statistics

For all statistical analyses, confirm that the following items are present in the figure legend, table legend, main text, or Methods section.

n/a	Confirmed	
☐	☒	The exact sample size (*n*) for each experimental group/condition, given as a discrete number and unit of measurement
☒	☐	A statement on whether measurements were taken from distinct samples or whether the same sample was measured repeatedly
☒	☐	The statistical test(s) used AND whether they are one- or two-sided<br>*Only common tests should be described solely by name; describe more complex techniques in the Methods section.*
☒	☐	A description of all covariates tested
☒	☐	A description of any assumptions or corrections, such as tests of normality and adjustment for multiple comparisons
☐	☒	A full description of the statistical parameters including central tendency (e.g. means) or other basic estimates (e.g. regression coefficient) AND variation (e.g. standard deviation) or associated estimates of uncertainty (e.g. confidence intervals)
☒	☐	For null hypothesis testing, the test statistic (e.g. *F*, *t*, *r*) with confidence intervals, effect sizes, degrees of freedom and *P* value noted<br>*Give P values as exact values whenever suitable.*
☒	☐	For Bayesian analysis, information on the choice of priors and Markov chain Monte Carlo settings
☒	☐	For hierarchical and complex designs, identification of the appropriate level for tests and full reporting of outcomes
☒	☐	Estimates of effect sizes (e.g. Cohen's *d*, Pearson's *r*), indicating how they were calculated

Our web collection on statistics for biologists contains articles on many of the points above.

Software and code

Policy information about availability of computer code

Data collection	No software was used for collection of data.
Data analysis	DIALS (current version is 3.11. 0) and XDS (current version: Jan 10, 2022) were used for processing of crystallographic data sets. Software within the CCP4i2 interface (current version: v2.0.03) was used for structure determination: MOLREP for molecular replacement (current version 11.9.02), COOT for model building (current version 0.9.8.5 EL), REFMAC for refinement (current version 5.8.0352), PRIVATEER for carbohydrate validation (current version MKIV). PDBePISA (version 1.48) was used to analyse protein-protein interfaces within crystal structures. UCSF ChimeraX (version 1.4) was used to make images depicted protein crystal structures. The PDB-REDO server (version 3.0.0; https://pdb-redo.eu/#MORE) was used to generate restraints for the IgG1 FcE382S model. MolProbity (current version: 4.2) and the PDB validation server (https://validate-rcsb-2.wwpdb.org) were used for model validation prior to deposition. Polder maps were calculated with the phenix.polder tool within the PHENIX software suite (version 1.20.1-4487-000).

For manuscripts utilizing custom algorithms or software that are central to the research but not yet described in published literature, software must be made available to editors and reviewers. We strongly encourage code deposition in a community repository (e.g. GitHub). See the Nature Portfolio guidelines for submitting code & software for further information.

Data

Policy information about availability of data

All manuscripts must include a data availability statement. This statement should provide the following information, where applicable:

- Accession codes, unique identifiers, or web links for publicly available datasets
- A description of any restrictions on data availability
- For clinical datasets or third party data, please ensure that the statement adheres to our policy

Structure factor files and atomic coordinates for each of the crystallographic models presented in this study have been deposited in the PDB, with accession codes 8A47 (https://www.rcsb.org/structure/8A47), 8A48 (https://www.rcsb.org/structure/8A48), and 8A49 (https://www.rcsb.org/structure/8A49), for the IdeSC94A-IgG1 FcE382A complex, IgG1 FcE382S and the EndoSD233A/E235L-IgG1 FcE382R complex, respectively. Previously-elucidated crystal structures analysed in this study are deposited in the PDB, with accession codes 3AVE (https://www.rcsb.org/structure/3AVE), 1Y08 (https://www.rcsb.org/structure/1Y08), 2AU1 (https://www.rcsb.org/structure/2AU1), 2AVW (https://www.rcsb.org/structure/2AVW) and 6EN3 (https://www.rcsb.org/structure/6EN3), which correspond to wild-type IgG1 Fc, IdeSC94S, wild-type IdeS, IdeSC94A and EndoSD233A/E235L, respectively.

Human research participants

Policy information about studies involving human research participants and Sex and Gender in Research.

Reporting on sex and gender	N/A for this research
Population characteristics	N/A for this research
Recruitment	N/A for this research
Ethics oversight	N/A for this research

Note that full information on the approval of the study protocol must also be provided in the manuscript.

Field-specific reporting

Please select the one below that is the best fit for your research. If you are not sure, read the appropriate sections before making your selection.

☒ Life sciences ☐ Behavioural & social sciences ☐ Ecological, evolutionary & environmental sciences

For a reference copy of the document with all sections, see nature.com/documents/nr-reporting-summary-flat.pdf

Life sciences study design

All studies must disclose on these points even when the disclosure is negative.

Sample size	Sample size is not applicable to the single-crystal X-ray crystallographic research because we are reporting novel crystal structures only. For the crystal counting experiment (Fig. 1a), crystallisation experiments were set up once only but repeated with different variants of the Fx proteins (see Replication section).
Data exclusions	No data were excluded from the analyses.
Replication	For the propensity to crystallise experiment, a single crystallisation experiment (i.e. one plate) was set up for each IgG1 Fc construct (wild-type and Fc E382S as depicted in Fig 1a, Fc E382A and Fc E382R as mentioned in the text). Experiments were not repeated; however, the three "less-crystallisable" IgG1 Fcs all showed similarly reduced crystallisation compared to the wild-type IgG1 Fc, which constitutes replications of the observation that mutation of residue E382 reduces the crystallisation ability of IgG1 Fc.<br>The analytical SEC traces and SDS-PAGE gel shown in supplementary Fig. 2 represent the single batches of protein complexes produced for crystallisation experiments (replication was not necessary since protein crystals were grown during this single attempt).
Randomization	Randomisation is not applicable to this study because we are reporting novel crystal structures only.
Blinding	Blinding is not applicable to this study because we are reporting novel crystal structures only.

Reporting for specific materials, systems and methods

We require information from authors about some types of materials, experimental systems and methods used in many studies. Here, indicate whether each material, system or method listed is relevant to your study. If you are not sure if a list item applies to your research, read the appropriate section before selecting a response.

Materials & experimental systems

n/a	Involved in the study
☐	☒ Antibodies
☐	☒ Eukaryotic cell lines
☒	☐ Palaeontology and archaeology
☒	☐ Animals and other organisms
☒	☐ Clinical data
☒	☐ Dual use research of concern

Methods

n/a	Involved in the study
☒	☐ ChIP-seq
☒	☐ Flow cytometry
☒	☐ MRI-based neuroimaging

Antibodies

Antibodies used	IgG1 Fc antibodies for crystallisation were obtained from cloning and expression of pFUSE-hIgG1-Fc vectors (InvivoGen). E382R/S/A mutations were introduced with site-directed mutagenesis, using primers designed by Eurofins Genomics.
Validation	Antibodies were sequenced by Eurofins Genomics prior to their use in crystallisation.

Eukaryotic cell lines

Policy information about cell lines and Sex and Gender in Research

Cell line source(s)	FreeStyle 293-F cell line (ThermoFisher) was used for antibody expression.
Authentication	Authentication was carried out by supplier (ThermoFisher).
Mycoplasma contamination	Cells tested negative for mycoplasma contamination.
Commonly misidentified lines (See ICLAC register)	No commonly misidentified cell lines were used in this study.
